# Antibiotic interceptors: Creating safe spaces for bacteria

**DOI:** 10.1371/journal.ppat.1006924

**Published:** 2018-04-19

**Authors:** Akshay Sabnis, Elizabeth V. K. Ledger, Vera Pader, Andrew M. Edwards

**Affiliations:** MRC Centre for Molecular Bacteriology and Infection, Imperial College London, London, United Kingdom; Nanyang Technological University, SINGAPORE

## Bacteria can resist antibiotics by sequestering them

Antibiotics have underpinned numerous advances including surgery, organ transplantation, and cancer chemotherapy. Therefore, the emergence of bacterial pathogens that are resistant to therapeutic antibiotics poses enormous challenges to modern medicine [1].

**Fig 1:**
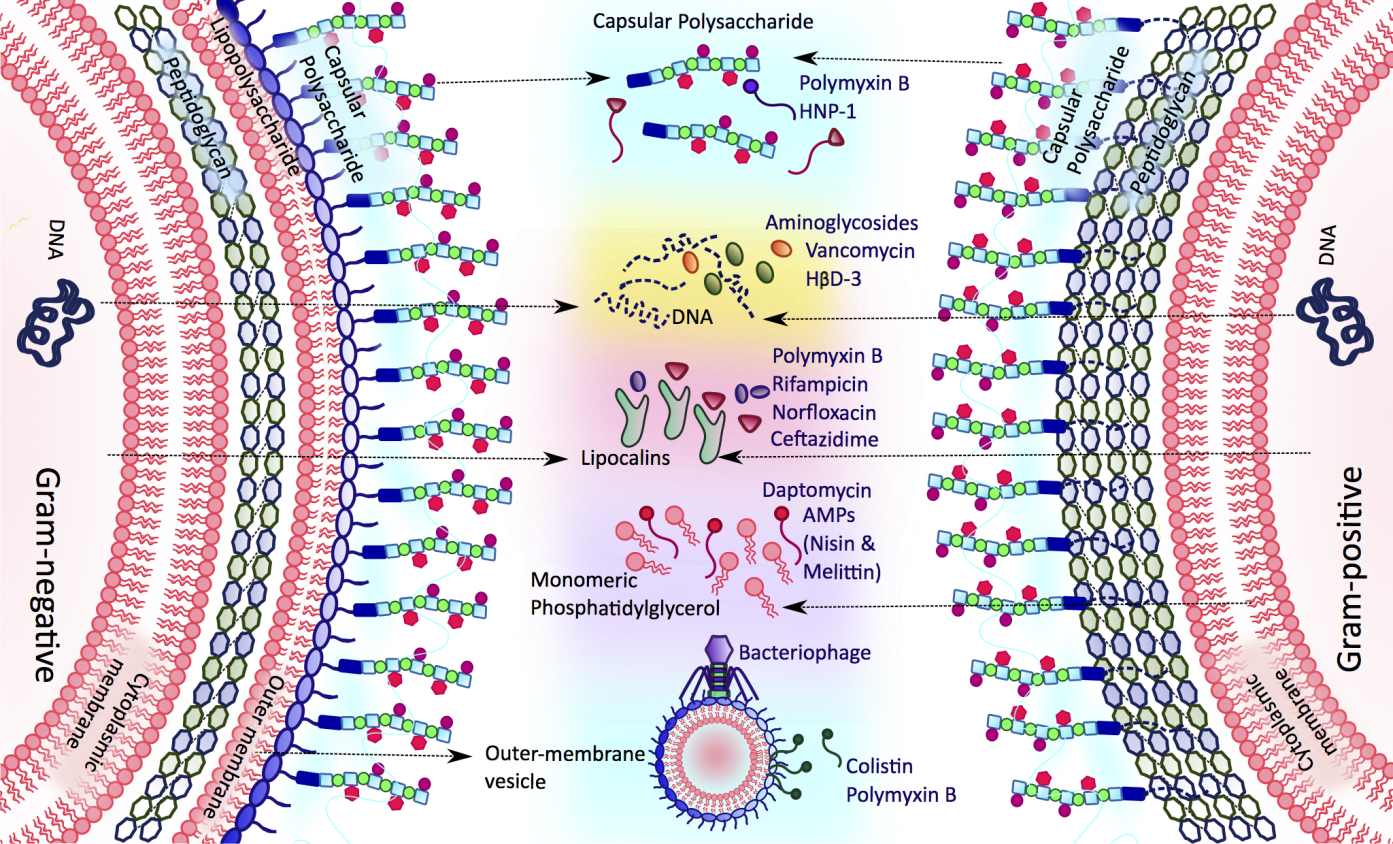
Bacteria release a diverse array of molecules to intercept antibiotics. AMP, antimicrobial peptide; HNP-1, human neutrophil protein-1; HβD-3, human β-defensin 3.

One of the key mechanisms by which bacteria resist antibiotics is the production of enzymes that inactivate antibacterial molecules via hydrolysis or chemical modification [1]. However, there is growing evidence that bacteria can also survive exposure to antibiotics by releasing molecules into the extracellular space that sequester the drug and prevent it from reaching its target. Some of these ‘antibiotic interceptors’ function as decoys by mimicking target molecules, whilst others share no obvious similarity to the target at all (Fig 1). Many interceptors also function as structural components of biofilms, thereby contributing to the antibiotic tolerance of these bacterial communities.

This article summarises our current understanding of antibiotic interceptors, explores the selection pressures for the emergence of these systems, and identifies future research avenues to characterise and overcome antimicrobial interception strategies.

## Membrane lipid decoys

Daptomycin and colistin are considered antibiotics of last resort, used to treat infections caused by highly drug-resistant gram-positive and gram-negative bacteria, respectively. They are both peptide antibiotics with functional similarity to cationic antimicrobial peptides (AMPs) found in the host. Daptomycin targets phosphatidylglycerol in the membrane of gram-positive bacteria, resulting in membrane depolarisation and bacterial death, whilst colistin binds to lipopolysaccharide (LPS) on the surface of gram-negative bacteria and disrupts membrane function [2,3].

Recent work has shown that gram-positive bacteria, including *Staphylococcus aureus* and *Enterococcus faecalis*, can evade daptomycin by releasing phosphatidylglycerol decoys from the membrane in response to the antibiotic [4,5]. These decoys sequester daptomycin, preventing the antibiotic from inserting into the bacterial membrane, and exist as monomers, indicating that their concentration does not exceed the critical micelle concentration [6]. The release of phospholipids occurs via a process that requires de novo lipid biosynthesis, demonstrating that this constitutes an active defence mechanism rather than simply arising as the result of damage to the membrane caused by the antibiotic [4,5]. However, neither the mechanism by which bacteria detect daptomycin nor the process by which monomeric phospholipids are released are understood.

Gram-negative bacteria also appear to employ decoys to survive exposure to membrane-targeting antimicrobials. Although the release of outer-membrane vesicles (OMVs) from gram-negative bacteria, such as *Escherichia coli* and *Pseudomonas aeruginosa*, occurs in the absence of antibiotic stress, the rate of vesicle production increases significantly in response to colistin or polymyxin B [7]. The released OMVs act as decoy receptors for these antibacterial agents, enabling bacteria to survive otherwise lethal concentrations of the antibiotics [7,8]. It is unknown whether the release of OMVs in response to membrane-targeting antibacterials is a regulated process or simply a consequence of membrane disruption. However, since OMV release can be regulated independently of membrane disruption, it is feasible that vesicle release in response to polymyxins forms part of a dedicated defence mechanism against these antibiotics [9].

## Antimicrobial interception by proteins

*Burkholderia cenocepacia* is a major cause of opportunistic lung infections, particularly in patients with cystic fibrosis, and is inherently resistant to many antibiotics. Upon exposure to bactericidal antibiotics, including polymyxin B, rifampicin, norfloxacin, and ceftazidime, *B*. *cenocepacia* releases a small protein known as a lipocalin, which sequesters the inducing antibiotic [10]. Once bound to the lipocalin, the antibiotic is unable to engage its target, enabling the bacterium to grow in the presence of otherwise inhibitory concentrations of the drugs [10]. The presence of lipocalin genes in several other pathogens, including *Mycobacterium tuberculosis* and *S*. *aureus*, raises the possibility that this constitutes a broadly conserved mechanism of antibiotic interception [10].

An additional mechanism by which proteins can become interceptors is via their release in OMVs. For example, proteases within OMVs were found to inactivate the AMP melittin [8]. Similarly, the presence of β-lactamases in membrane vesicles released from *Moraxella catarrhalis* or *S*. *aureus* protects both the producer and drug-sensitive bystander bacteria from β-lactams by hydrolysing the antibiotic [11,12].

## Antimicrobial interception by polysaccharides

Many pathogenic bacteria are enclosed within a polysaccharide capsule, which protects the cell from host immune defences, including phagocytosis, complement peptides and AMPs such as human neutrophil protein-1 (HNP-1), or the cathelicidin LL-37 [13,14]. However, exposure of *Klebsiella pneumoniae*, *Streptococcus pneumoniae*, or *P*. *aeruginosa* to the peptide antibiotic polymyxin B or HNP-1 triggers the release of polysaccharide from the bacterial surface [15]. It is unknown whether capsule release is due to damage caused by the antimicrobials or a specific bacterial response to stress, but this phenomenon promotes bacterial survival by sequestering polymyxin B and HNP-1 [15]. Furthermore, exposure of the opportunistic pathogen *Acinetobacter baumanii* to chloramphenicol or erythromycin results in hyperproduction of capsular exopolysaccharide, which confers resistance to these antibiotics, although the regulation and mechanism of this process is unclear [16].

In addition to capsular polysaccharide, other polysaccharides frequently provide an important structural component of biofilms, where they can also modulate susceptibility to antimicrobials. For example, the Psl exopolysaccharide contributes to the tolerance of *P*. *aeruginosa* biofilms to colistin and tobramycin, most likely by sequestering the antibiotics, as observed for capsular polysaccharides [17].

## Antimicrobial sequestration by DNA

Like exopolysaccharides, extracellular DNA (eDNA) is a major structural component of biofilms, as well as sequestering positively charged antimicrobials via electrostatic interactions [18]. For example, *P*. *aeruginosa* biofilms rich in eDNA can sequester aminoglycosides such as tobramycin, leading to increased bacterial survival [19]. Similarly, *Staphylococcus epidermidis* biofilms exposed to subinhibitory concentrations of vancomycin contain higher levels of eDNA than unexposed biofilms, although the regulatory and mechanistic basis for this is unknown [20]. This eDNA binds vancomycin, impeding its penetration through the biofilm, leading to increased bacterial survival [20]. In addition to antibiotics, eDNA also binds human β-defensin 3, a cationic host defence AMP, reducing its ability to kill both planktonic and biofilm forms of *Haemophilus influenzae* [21].

## What has driven the evolution of antibiotic interceptors?

Many bacteria exist in single or polymicrobial biofilms that are maintained by extracellular polymeric substances such as DNA, polysaccharides, proteins, and lipids [18]. Therefore, the evolution of biofilm formation may have provided the mechanisms used in interceptor production and release. Subsequently, it is likely that intermicrobial competition selected for the use of extracellular products as antibiotic interceptors. Whilst antibiotic resistance is a recent clinical problem, the underlying mechanisms are ancient, reflecting the presence of antibiotic-producing fungi and bacteria in the environment [22]. Bacteriophages are also prevalent in the environment and may, therefore, have contributed the emergence of OMVs as an extracellular defence mechanism [7].

In the context of polymicrobial biofilms, antibiotic interceptors become ‘public goods’, a shared resource between the bacteria that produce them and other cells that do not. This shared-goods approach may reduce the overall cost of producing interceptors whilst maintaining a high level of protection [17].

In addition to the threats from antibiotic-producing competitors and bacteriophages, pathogens must also overcome host defences. Since many of the interceptors described above protect bacteria from AMPs as well as antibiotics, the host immune system may have provided further selection pressure for the evolution of antibiotic interceptors [4,7,8,13,14,21,23]. Therefore, antibiotic interceptors may have evolved to provide bacteria with an innate defence against the broad spectrum of threats posed by competitors, bacteriophages, and host defences.

## Summary and future directions

The frequent inability of apparently appropriate antibiotic therapy to clear bacterial infections suggests that mechanisms beyond dedicated antibiotic resistance modulate treatment outcomes. One such mechanism involves the release of molecules into the extracellular space that sequester antibiotics before they can engage with their target. These recently discovered processes underscore the diversity of mechanisms by which pathogens have evolved resistance to both host defences and antibiotics (Fig 1, Table 1). However, whilst we are beginning to identify and characterise antibiotic interceptors, several major questions remain (Table 2). In particular, our lack of understanding of these mechanisms has made it difficult to fully assess their contribution to treatment failure. For example, the lack of mutants defective for capsule release or OMV production prevents appropriate testing of antibiotic susceptibility in vivo. Challenges in studying interceptors may be resolved using small-molecule inhibitors. For example, it has been shown that daptomycin-induced phospholipid release can be partially inhibited by the β-lactam antibiotic oxacillin [4]. In addition to determining the contribution of antibiotic interception to treatment failure, this approach may also improve treatment outcomes by identifying adjunctive therapeutic approaches that block the production of antimicrobial-sequestering molecules. Such an approach may also disrupt biofilm formation since many interceptors are important components of these structures [18]. In addition, since interceptors do not chemically inactivate antimicrobials, it may be possible to develop adjunctive therapeutic strategies that prevent or reverse antibiotic sequestration. For example, lipocalins could not sequester antibiotics when fat-soluble vitamins were present [10]. Finally, since several antibiotic interceptors also confer resistance to AMPs, inhibitors of interceptor production or function may contribute to treatment success by increasing the susceptibility of pathogens to host defences.

**Table 1:** Summary of antibiotic interceptors.

Class of Interceptor	Mechanism of Interception	Bacterial Species	Antimicrobials Sequestered	Reference
Lipid	Release of monomeric membrane phospholipids	*S*. *aureus*	Daptomycin, nisin, melittin	[4]
		*E*. *faecalis* *Streptococcus* spp.	Daptomycin	[5]
	Release of OMVs	*E*. *coli*	Polymyxin B, colistin	[6],[7]
	Transport of hydrolytic enzymes within OMVs	*E*. *coli*	Melittin	[8],[9]
		*M*. *catarrhalis*	Amoxicillin	[11]
Protein				
	Release of lipocalins	*B*. *cenocepacia*	Rifampicin, norfloxacin, ceftazidime, polymyxin B	[10]
Polysaccharide	Release of free capsular polysaccharide from bacterial surface	*P*. *aeruginosa* *S*. *pneumoniae* *K*. *pneumoniae*	HNP-1, polymyxin B	[13–17]
DNA	Release of eDNA in biofilms	*P*. *aeruginosa*	Tobramycin, gentamicin	[19]
		*S*. *epidermidis*	Vancomycin	[20]
		*H*. *influenzae*	Human beta-defensin-3	[21]

**Abbreviations:** eDNA, extracellular DNA; HNP-1, human neutrophil protein-1; OMVs, outer membrane vesicles.

**Table 2:** Outstanding questions.

**Outstanding Questions:**
1. What is the contribution of antibiotic interceptors to clinical treatment failure?
2. How do bacteria sense the presence of antibiotics and AMPs?
3. How do bacteria release interceptors?
4. Can we block the production of interceptors to promote treatment outcomes?

**Abbreviation:** AMP, antimicrobial peptide.
